# Lactobacillus Sps in Reducing the Risk of Diabetes in High-Fat Diet-Induced Diabetic Mice by Modulating the Gut Microbiome and Inhibiting Key Digestive Enzymes Associated with Diabetes

**DOI:** 10.3390/biology10040348

**Published:** 2021-04-20

**Authors:** Aneela Gulnaz, Jawad Nadeem, Jong-Hun Han, Lee-Ching Lew, Jae-Dong Son, Yong-Ha Park, Irfan A. Rather, Yan-Yan Hor

**Affiliations:** 1 Department of Biotechnology, Yeungnam University, 280 Daehak-Ro, Gyeongsan, Gyeongbuk 38541, Korea; aneelamicro@gmail.com (A.G.); jawadnadeem04@gmail.com (J.N.); rtjh0307@daum.net (J.-H.H.); peter@ynu.ac.kr (Y.-H.P.); 2 Probionic Corp. Jeonbuk Institute for Food-Bioindustry, 111-18, Wonjangdong-gil, Deokjin-gu, Jeonju-si, Jeollabuk-do 38541, Korea; lewleeching@gmail.com; 3 Department of Veterinary Medicine, College of Veterinary Medicine, Gyeongsang National University, Jinju-si, Gyeongsangnam-do 52828, Korea; beeast0070@gmail.com; 4 Department of Biological Sciences, Faculty of Science, King Abdulaziz University, Jeddah 21589, Saudi Arabia

**Keywords:** Type 2 diabetes, gut-microbiome, HFD-diet, *Lactobacillus*, probiotics, α-glucosidase, α-amylase, 16S RNA gene, mice, fecal samples

## Abstract

**Simple Summary:**

Type 2 diabetes (T2D) is increasingly spreading across the globe. The disease is linked to a disruption of gut microbiome. Probiotics are essential gut microbiota modulators proven to restore microbiota changes, thereby conferring health to its host. This study aimed to use probiotics (lactobacilli) and their metabolites as natural anti-diabetic therapy through the modulation of gut microbiota and inhibit diabetes-causing enzymes. Lactobacillus-treated high-fat diet mice showed lower blood glucose levels and body weight. Interestingly, our study also proved that the lactobacilli altered gut microbiota composition by suppressing opportunistic bacteria that are highly associated with metabolic diseases. Our findings substantiate the use of probiotics as natural anti-diabetic therapeutics.

**Abstract:**

Obesity caused by a high-fat diet (HFD) affects gut microbiota linked to the risk of type-2 diabetes (T2D). This study evaluates live cells and ethanolic extract (SEL) of *Lactobacillus sakei* Probio65 and *Lactobacillus plantarum* Probio-093 as natural anti-diabetic compounds. In-vitro anti-diabetic effects were determined based on the inhibition of α-glucosidase and α-amylase enzymes. The SEL of Probio65 and Probio-093 significantly retarded α-glucosidase and α-amylase enzymes (*p* < 0.05). Live Probio65 and Probio-093 inhibited α-glucosidase and α-amylase, respectively (*p* < 0.05). In mice fed with a 45% kcal high-fat diet (HFD), the SEL and live cells of both strains reduced body weight significantly compared to HFD control (*p* < 0.05). Probio-093 also improved blood glucose level compared to control (*p* < 0.05). The gut microbiota modulatory effects of lactobacilli on HFD-induced diabetic mice were analyzed with qPCR method. The SEL and live cells of both strains reduced phyla *Deferribacteres* compared to HFD control (*p* < 0.05). The SEL and live cells of Probio-093 promoted more *Actinobacteria* (phyla), *Bifidobacterium*, and *Prevotella* (genus) compared to control (*p* < 0.05). Both strains exerted metabolic-modulatory effects, with strain Probio-093 showing more prominent alteration in gut microbiota, substantiating the role of probiotics in gut microbiome modulations and anti-diabetic effect. Both lactobacilli are potential candidates to lessen obesity-linked T2D.

## 1. Introduction

Diabetes has become a global problem in recent years, and it is closely related to obesity. Obesity originates from lifestyle changes and diets that include high-fat contents [1]. Currently, the issue of concern is about the obesity caused by a high-fat diet (HFD) and its effect on the gut microbiota. One of the striking models gaining increased attention is the HFD-induced diabetic model in mice. The high fat feeding model contributes to obesity, hypertriglyceridemia, inflammation, lack of beta cell compensation, and distorted glucose homeostasis [2]. An individual who is overweight has more chances of being affected with type 2 diabetes (T2D). Excess body fat is deposited around the cells and develops insulin resistance, and it is a sign of T2D [3]. When mice are fed with high fat diet for several weeks, their weight increases dramatically, and glucose level upraises. Hyperglycemia (high glucose level in the blood) usually develops within four weeks by continuous consumption of an HFD [4]. HFD’s excess contents of fats and carbs are the primary source of diabetes. These complex carbohydrates and starch are converted into glucose with enzymes, like α-glucosidase (α-GLU) and α-amylase in the gastrointestinal tract. These changes lead to increased glucose absorption by the intestine, and the level of blood glucose rises [5]. Based on this principle, inhibition of the enzymes α-amylase and α-GLU could help glycemic control, thereby facilitating the usage of these enzyme inhibitors as diabetic control drugs. Acarbose, miglitol, and voglibose are synthetic therapeutic antagonists that are selective against postprandial hyperglycemia [6]. As a competitive inhibitor, acarbose reduces α-GLU and α-amylase in the small intestine’s brush line, delaying glucose absorption by reducing complex carbohydrate breakdown [7,8]. Acarbose is a US Food and Drug Administration approved oral anti-diabetes drug and also serves as a gold standard in screening for potential inhibitors of the carbohydrate hydrolase enzymes as anti-diabetic drug [9]. However, these synthetic inhibitors are accompanied with some gastrointestinal side effects, including diarrhea and bloating, prompting the search of new drugs with similar potency, and better safety profile [10].

Compelling evidence supports that the intestinal microbiome is important in the pathophysiology of T2D. The gut microbiota is a microbial population in the internal surface area of the gastrointestinal tract. Gut microbes contribute to human health through polysaccharide breakdown, acid bile modification, assimilation of nutrients, intestine permeability, and inflammatory reactions. Alterations in the gut microbial community may be linked to weight gain and insulin resistance [11]. Studies on germ-free mice showed that intestinal microorganisms are essential to maintain immune and gastrointestinal functions, the gut mucosal barriers, structural integrity, xenobiotic and drug metabolism, and protection against pathogens [12,13,14,15]. In the immune system, the gut microbiota has epigenetic control, regulating the differentiation of regulatory T cells (Tregs) by butyrate (SCFA). Without microbiota, intestinal mucosal immunity is not developed. Individuals exhibit slighter mesenteric lymph nodes, PP (Peyer’s patches, gut-associated lymphoid tissue), and lessened extent of immune cells such as IgA-generating plasma cells, CD4^+^ LP (lamina propria) T-cells and intraepithelial αβ T-cell receptor CD8^+^ cells. As a result, animals would have a discounted stability to fend off pathogens [16]. The pattern recognition receptors (PRRs) help locate pathogens in the host, promoting the emission of innate effector molecules after detecting of conserved molecular structures known as pathogen-associated molecular patterns. As regarding gut microbiota, through PRRs, can regulate the formation of AMPs (production of α-defensins, β-defensins, and other bactericidal substances termed antimicrobial peptides (AMPs)) and also control the expression of genes comprised in inflammatory and pain responses [17,18]. The microbiota releases various cellular factors that affect human metabolism due to their ability to interact with receptors on epithelial and subepithelial cells [19]. The gut microbiota receives its nutrients from ingested dietary components as well as host-derived components such as shed epithelial cells and mucus.

Consequently, microbiota generates many metabolites like SCFA and several molecules, such as vitamin K and vitamin B constituents, that affect human health and metabolism [20,21]. Type 2 diabetes mellitus, on the other hand, is an oxidative stress disorder which is also contributed to by a deficit in vitamin B and folic acid deficits [22]. As a result of this connection, vitamin B12 deficiency may potentially be considered a risk factor for diabetic complications [23]. Peripheral neuropathy is one of the most common complications of type 2 diabetes mellitus [24]. Vitamin K consumption has been linked to insulin sensitivity, glucose tolerance, and thereby diabetes in many studies [25]. For example, the PREDIMED research in Spain looked at dietary vitamin K intake and diabetes indicators [26]. After a year of follow-up, those with the highest intakes had lower plasma concentrations of ghrelin, glucose-dependent insulinotropic peptide, glucagon-like peptide-1, IL-6, leptin, TNF, and visfatin. Increased vitamin K intakes were linked to a lower risk of diabetes mellitus in the same study [27].

Accordingly, the gut microbial composition variations are responsible for energy availability, calories storage in adipose tissues, and metabolic disorders such as obesity and diabetes [28]. Furthermore, developing facts suggest that the intestinal microbes represent a key factor in calculating the host response to nutrients. These microbes change the food components and generate numerous useful extracted metabolites that affect the host’s health and physiology in numerous ways [29]. A number of chronic gastrointestinal diseases that have been conjoined with changes in gut microbial community include irritable bowel syndrome (IBS) and systemic disorders such as T2D and obesity, as well as the onset of colorectal cancer [30].

Probiotics can restore the gut microbiome composition and introduce beneficial functions to gut microbial communities, leading to improvement or prevention of inflammation of the gut and other intestinal diseases. In 2011, experts of the Food and Agriculture Organization of the United Nations (FAO) and the World Health Organization (WHO) stated probiotics as “live microorganisms which give rise health benefits on the host when supplied in adequate quantity”. Products containing probiotic bacteria have been progressively applied to treat or prevent several metabolism-associated disorders such as obesity, T2D, inflammatory bowel disease, discrete types of diarrhea, intestinal infections, irritable bowel syndrome, chronic idiopathic constipation, and some other respiratory, allergic, and pulmonary diseases [31]. Research in animals and people suggests that a probiotic supplement may regulate gut microbiota, thereby improving the prognosis for many metabolic disorders, including diabetes [32]. The probiotics mechanism includes regulation of microbial intestinal populations, pathogen destruction, immunomodulation, activation and differentiation of epithelial cell proliferation, and fortification of intestinal barrier [33]. Intestinal microbiota modulation by probiotics appears to offer beneficial outcomes to insulin-resistant individuals via mechanisms both related and unrelated to inflammation.

Moreover, diverse evidence shows that the consumption of probiotics may be associated with lowering blood glucose and cholesterol, reducing the inflammatory response and anti-tumor effects via their surface polysaccharides and metabolites of short-chain fatty acid (SCFAs) [34]. Probiotic *Lactobacillus* strains enhance the integrity of the intestinal barrier, which may result in the maintenance of immune tolerance, decreased translocation of bacteria across the intestinal mucosa, and disease phenotypes including gastrointestinal infections, IBS, and IBD [35].

This study underpins to determine the in-vitro anti-diabetic effect of *L. sakei* Probio65 and *L. plantarum* Probio-093 via the inhibition of α-GLU and α-amylase as well as the changes in gut microbial diversity of HFD induced diabetes mice.

## 2. Materials and Methods

### 2.1. Culture of Bacterial Strains

Two strains of lactic acid bacteria (LAB), *Lactobacillus sakei* Probio65 and *Lactobacillus plantarum* Probio-093, were obtained from the culture collection of microbiome lab in Yeungnam University, and they were previously isolated from famous Korean fermented food, kimchi. Stock cultures were preserved in 60% glycerol (−20 °C) and were activated in sterile de Mann, Rogosa, Sharpe (MRS) (MB cell, Kisan Bio, Seoul, Korea) broth and incubated at 37 °C for 24 h.

### 2.2. Preparation of SEL

The preparation of SEL was carried out following the same protocol as reported in our previous studies [36,37]. Briefly, the fresh culture of *Lactobacillus* strains was mixed with an equal volume of 95% ethanol, and after constant shaking at 150 rpm for 1 h, was centrifuged at 10,000 g for 5 min at 4 °C. The supernatant was collected and brought to a viscous pallet by vacuum. The dried sediment was suspended in sterile phosphate buffer saline (PBS, pH 7.4).

### 2.3. α-Amylase Inhibition

A modified method of α-amylase activity determined by Kusano et al. (2011) was applied [38]. Porcine pancreas α-amylase was acquired from Sigma (St. Louis, MO, USA). The soluble starch was implemented as a substrate and directed by boiling 1% in distilled water for 5 min, and then cooled down to room temperature. The sample (80 µL) and substrate (80 µL) were mixed in 100 µL of 0.1M PBS. After that, 40 µL of 2U/mL α-amylase solution was supplemented, and this reaction solution was incubated at 37 °C for 30 min. The reaction was ceased by adding 50 µL 0.1M HCl; before that that 3 µL of the 0.3 mg/mL iodine solution was reacted and then incubated at 37 °C for 5 min. The absorbance was quantified at 620 nm by a microplate reader (Sunrise Tecan, Switzerland). The α-amylase inhibition was expressed as the percentage of enzyme inhibition using the equation (Equation (1)).

(1) $$\mathrm{Inhibition}\;\left(\%\right)=\left[\frac{\mathrm{control}\;\mathrm{absorbance}-\mathrm{sample}\;\mathrm{absorbance}}{\mathrm{control}\;\mathrm{absorbance}}\right]\times 100$$

### 2.4. α-Glucosidase Inhibition

Inhibition of α-GLU by probiotics was performed according to the method as defined by Kim et al. (2011) by using α-GLU from Saccharomyces cerevisiae (Sigma, USA) with a few modifications [39]. Firstly, α-GLU (25 µL, 0.17 U/mL) and potassium phosphate buffer (50 µL) were intermixed with 10 µL of the test sample. PBS and MRS were used as a control group. Preceding incubation at 37 °C for 30 min, 5mM p-nitrophenol-α D-glucopyranoside (pNPG, 25 µL) was added. The enzymatic reaction was allowed to continue and was halted by the inclusion of 100 µL of 0.2 M Na2CO3. The absorbance was examined at 405 nm using a microplate. The effect of the strains on the α-GLU inhibition was calculated as the percentage of enzyme inhibition by using the Equation (1).

### 2.5. Animal Model and Diet

A total of forty C57BL/6J male mice (4 weeks old) were obtained from the animal facility center of Yeungnam University. Mice cages were kept at a consistent temperature and 12 h light/dark cycle at relative humidity of 60–70%. Animals handling and management approval for the research was approved by the Animal Ethical Committee of the Yeungnam University (ethics approval number 2018-037), Gyeongsan, Republic of South Korea. Animals were divided into eight groups, and each group consisted of 5 mice (*n* = 5). Mice were acclimatized for one week. All groups were fed with a 45% kcal high-fat diet (D12451) (OpenSource Diets, New Brunswick, NJ, USA) to induce obesity and T2D except the naïve group (diet formulation is mentioned in Table 1). Groups were categorized like; (1): Naïve (normal diet), (2): HFD (high-fat diet), (3): metformin (HFD, receiving drug metformin (0.25 mg/g/day)), (4): MRS (HFD, receiving de Mann, Rogosa, Sharpe SEL (5 µL/g/day)), (5): Probio65 Live cells (HFD, receiving *Lactobacillus sakei* Probio65 (10⁸ CFU/day)), (6): Probio-093 Live cells (HFD, receiving *Lactobacillus plantarum* Probio-093 (10⁸ CFU/day)), (7): Probio65 SEL (receiving *Lactobacillus sakei* Probio65 (5 µL/g/day)) and (8): Probio-093 SEL (HFD, receiving *Lactobacillus plantarum* Probio-093 (5 µL/g/day)). Mice were sacrificed after 8 weeks of treatment, and then all samples were stored in a nitrogen tank until further processing.

### 2.6. Bodyweight and Glucose Measurement

The body weight change, feed intake, as well as glucose levels were observed after every two weeks. For the glucose measurement, one drop of blood was collected from the tail of mice and checked using a blood glucose test strip (Accu-Chek, IN, USA).

### 2.7. DNA Extraction from Fecal Pellets

Fecal samples were acquired after eight weeks of treatment. The fecal samples were stored in a nitrogen tank and subsequently freeze-dried overnight. Approximately 100 mg-200 mg freeze-dried fecal samples were used for DNA extraction according to the method as described in the stool DNA extraction kit (Bioneer, Daejeon, Korea). The absorbance ratios of 260/280 nm and 260/230 nm were checked to assess and quantify the purity of DNA samples using a micro-spectrophotometer (Allsheng, HGH, Hangzhou, China).

### 2.8. Microbiome Analysis Using 16S rRNA Gene Target Primers with RT qPCR

A set of designed primers targeting 16S rRNA gene belonging to different taxa at phylum level including *Bacteroidetes*, *Firmicutes*, *Actinobacteria*, *Verrucomicrobia*, *Tenericutes*, *Deferribacteres*, and *Proteobacteria*; and at the genus level *Lactobacillus*, *Bacteroides*, *Prevotella*, *Bifidobacterium*, *Desulfovibrio*, and *Clostridium* were used to quantify the predominant microbial communities in the gut of mice (Table 2).

### 2.9. Primers Efficiencies

A standard procedure for primers amplification efficiencies was determined by making serial dilutions of total fecal DNA. Consequently, by considering the linear regression based on C_T_ values, the values of efficiency were gained from the slope of the line (Table 3). Dilution points were tested thrice. Non-template control was also inspected in each assay.

Using total DNA, the amount of each bacterial taxa was quantified with the ABI StepOne Plus Real-time PCR system (Applied Biosystem, Foster City, CA, USA). qPCR conditions were 95 °C for 10 min, followed by 40 cycles of 95 °C for 15 s and 60 °C for 1 min. The qPCR mixture contained 5 µL of 2xFastStart SYBR green with dye1, 0.5 µL of forward and reverse primer (total concentration 10 pmol), and 1 µL of template DNA. The obtained CT values from each primer pair first transformed into average and then converted into percentage by using the Equation (2):

(2) $$x=\frac{{\left(\mathrm{Eff}.\;\mathrm{Univ}\right)}^{\mathrm{CT}\;\mathrm{univ}}}{{\left(\mathrm{Eff}.\;\mathrm{Spec}\right)}^{\mathrm{CT}\;\mathrm{spec}}}\times 100$$

In the above formula, Eff.Univ determines the efficiency of universal primer (2 = 100% and 1 = 0%) and Eff.Spec is the efficiency of taxon-specific primers. CT univ and CT spec are the C_T_ values obtained from thermocycler. The attained X value represents the percentage of 16S taxon-specific bacteria in a sample. These acquired C_T_ values, after fecal samples are analyzed with qPCR measure, were utilized to calculate the extent of higher bacterial taxa within the feces.

### 2.10. Statistical Analysis

All results are presented as mean ± S.E.M. Statistical analysis was carried out by GraphPad Prism 6.0 software (GraphPad, San Diego, CA, USA). The one-way ANOVA was used to study the significant differences between the control group mean, and all samples mean with one variable. The mean of multiple groups with two independent variables was measured by two-way ANOVA. To compare the mean of only two groups, an unpaired t-test was used. The *p*-values less than 0.05 were dignified as statistically significant.

## 3. Results

### 3.1. Enzyme Inhibition Assays

**Figure 1:**
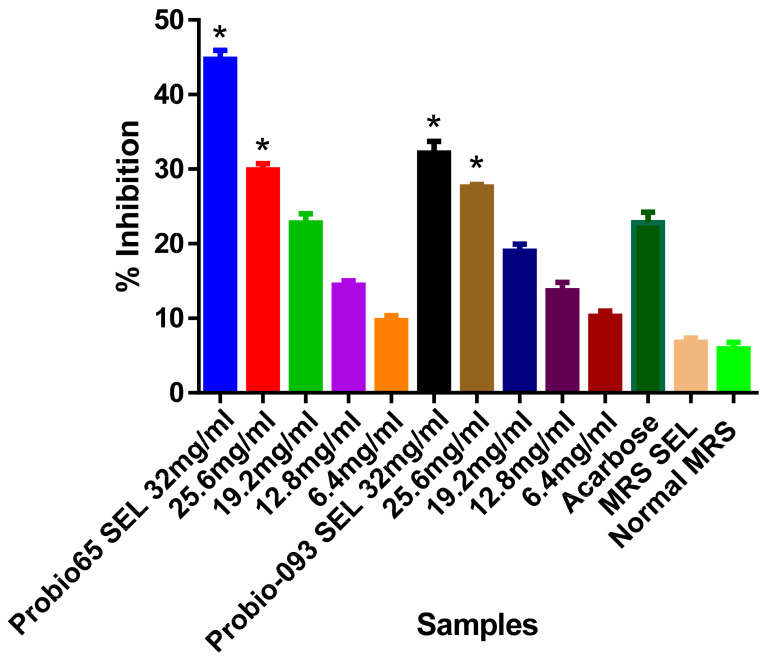
Inhibition of α-glucosidase activity with different concentrations of ethanolic extract (SEL) (6.4–32 mg/mL) of Probio65 (*L. sakei* Probio65) and Probio-093 (*L. plantarum* Probio-093). Acarbose was used as a reference indicator. MRS SEL and normal MRS were used as blank controls. Results were expressed as the mean ± S.E.M of triplicate data in a single experiment. Data was analyzed using one-way ANOVA. * Indicate significant at *p* < 0.05 when compared to the acarbose.

The α-GLU inhibition results were illustrated in Figure 1. In our results, the highest marked inhibition was 45% by 32 mg/mL of SEL of *L. sakei* Probio65 for α-GLU as compared to acarbose (*p* < 0.05). Alternate concentration 25.6 mg/mL of SEL of *L. sakei* Probio65 also considerably hindered this enzyme (*p* < 0.05). In the same figure, 32 mg/mL SEL of *L. plantarum* Probio-093 restricted the activity of α-GLU by 35% (*p* < 0.05). The 25.6 mg/mL of SEL of *L. plantarum* Probio-093 also showed notable obstruction, 28% as opposed to α-GLU (*p* < 0.05). Significant inhibition was not observed between acarbose and other concentrations (19.2mg/mL, 12.8mg/mL, and 6.4mg/mL).

**Figure 2:**
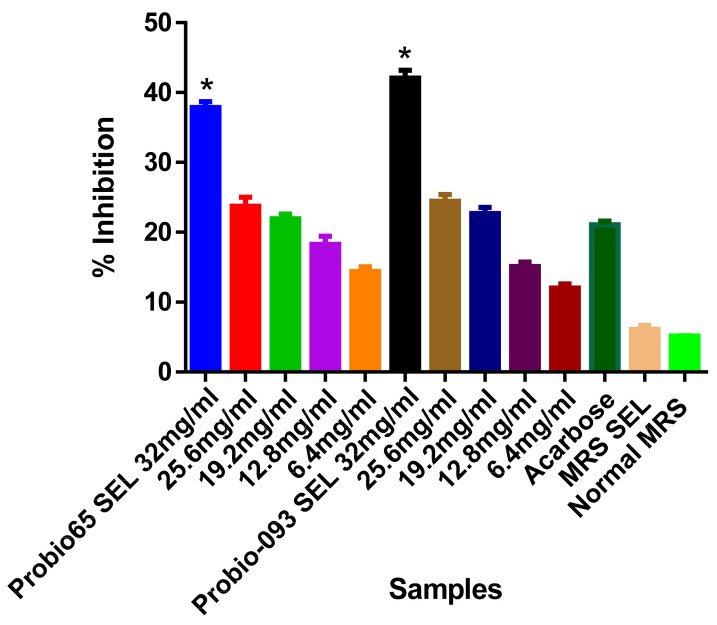
Inhibition of α-amylase activity with different concentrations of ethanolic extract (SEL) (6.4–32 mg/mL) of Probio65 (*L. sakei* Probio65) and Probio-093 (*L. plantarum* Probio-093). Acarbose was used as a reference indicator. MRS SEL and normal MRS were used as blank control. Results were expressed as the mean ± S.E.M of triplicate data in a single experiment. Data was analyzed using one-way ANOVA. * Indicate significant at *p* < 0.05 when compared to the acarbose.

As shown in Figure 2, based on the a-amylase inhibition test, the ethanolic extract of *L. sakei* Probio65 showed remarkable enzyme hindrance activity 38% with a concentration of 32 mg/mL (*p* < 0.05). The impeding effect of SEL of *L. plantarum* Probio-093 was 43% with 32 mg/mL (*p* < 0.05). No significant changes were examined between acarbose and concentrations from 25.6 mg/mL to 6.4 mg/mL. However, the rate of retardation could be raised at the increased SEL samples concentrations.

**Figure 3:**
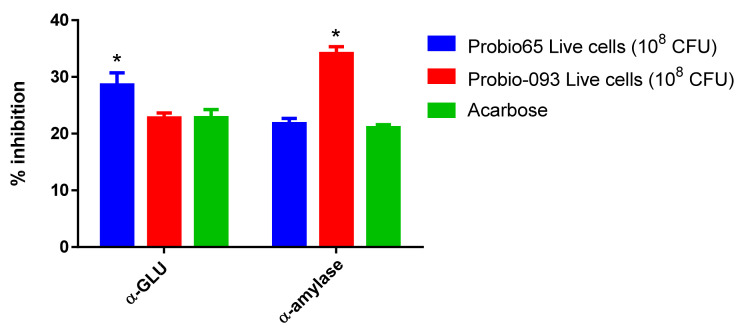
Inhibition of α-glucosidase and α-amylase activity after treatment with live cells of *L. sakei* Probio65 and *L. plantarum* Probio-093. Results were expressed as the mean ± S.E.M of triplicate data in a single experiment. Statistical analysis was performed with two-way ANOVA. * Indicate significant at *p* < 0.05 when compared to the acarbose.

According to Figure 3, the suppression of α-GLU and α-amylase was also studied with live cells of both tested strains *L. sakei* Probio65 and *L. plantarum* Probio-093. The Live cells (10⁸ CFU) of *L. plantarum* Probio-093 showed 35% considerable repression against α-amylase (*p* < 0.05), whereas α-GLU enzyme activity was not resisted. The live cells of *L. sakei* Probio65 significantly suppressed 30% α-GLU enzyme (*p* < 0.05), but no significant effect was seen on α-amylase inhibition when contrasted with acarbose.

### 3.2. Bodyweight and Glucose Level

**Figure 4:**
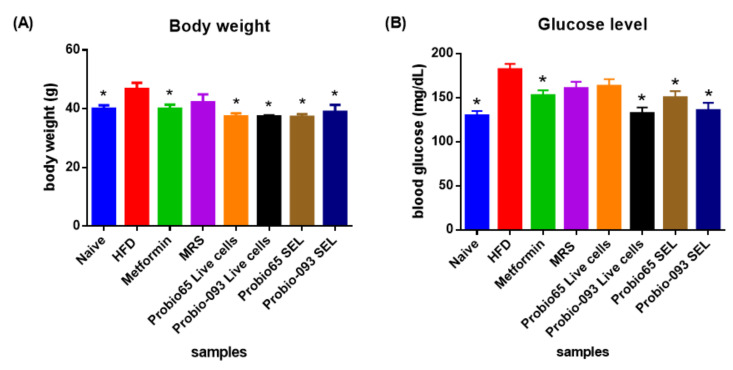
(**A**) Changes in body weight and (**B**) glucose level of mice after 8 weeks of high-fat diet treatment. Naïve: untreated mice fed with normal diet (*n* = 5); HFD: untreated mice fed with high-fat diet containing 45 kcal% fat (*n* = 5); Metformin: mice on high-fat diet (45 kcal% fat) and gavaged with metformin (0.25 mg/g/day); MRS: mice on high-fat diet (45 kcal% fat) and gavaged with De Man, Rogosa and Sharpe; Probio65 Live cells: mice on high-fat diet (45 kcal% fat) and gavaged with live *Lactobacillus sakei* Probio65 (10⁸ CFU/day); Probio-093 Live cells: mice on high-fat diet (45 kcal% fat) and gavaged with live *Lactobacillus plantarum* Probio-093 (10⁸ CFU/day); Probio65 SEL: mice on high-fat diet (45 kcal% fat) and gavaged with ethanolic extract of *Lactobacillus sakei* Probio65 (5 µL/g/day); Probio-093 SEL: mice on high-fat diet (45 kcal% fat) and gavaged with ethanolic extract of *Lactobacillus plantarum* Probio-093 (5 µL/g/day). The results are expressed as mean ± S.E.M. Statistical analysis was performed with one-way ANOVA. * Indicate significant at *p* < 0.05 when compared to the HFD.

Figure 4 reveals changes in glucose level and body weight. The body weight and glucose level of all mice significantly decreased over the experimental period when compared with HFD group mice upon 8 weeks.

As displayed in Figure 4A, the HFD group increased 15% more body weight as correlated with naïve group (*p* < 0.05). Mice administered with metformin drug and *L. plantarum* Probio-093 SEL displayed 15% lower body weight, similar to the naïve group (*p* < 0.05). Mice that received *L. sakei* Probio65 Live cells and SEL featured more reduction in the body weight, i.e., 16% compared to the HFD group (*p* < 0.05). Among all groups, *L. plantarum* Probio-093 Live cells mice group presented the highest markdown, 17.3% in body weight (*p* < 0.05).

Figure 4B depicted that the mice that received HFD showed a 38.5% elevated blood glucose level, reaching 180 mg/dL compared to naïve mice, which were only 130 mg/dL. Mice treated with *L. plantarum* Probio-093 Live cells indicated the most significant reduction (44%) in blood glucose level in comparison to the HFD mice, followed by *L. plantarum* Probio-093 SEL (28.6%), with metformin and *L. sakei* Probio65 SEL both 20% (*p* < 0.05).

### 3.3. Gut Microbiota Composition at the Phylum Level

**Figure 5:**
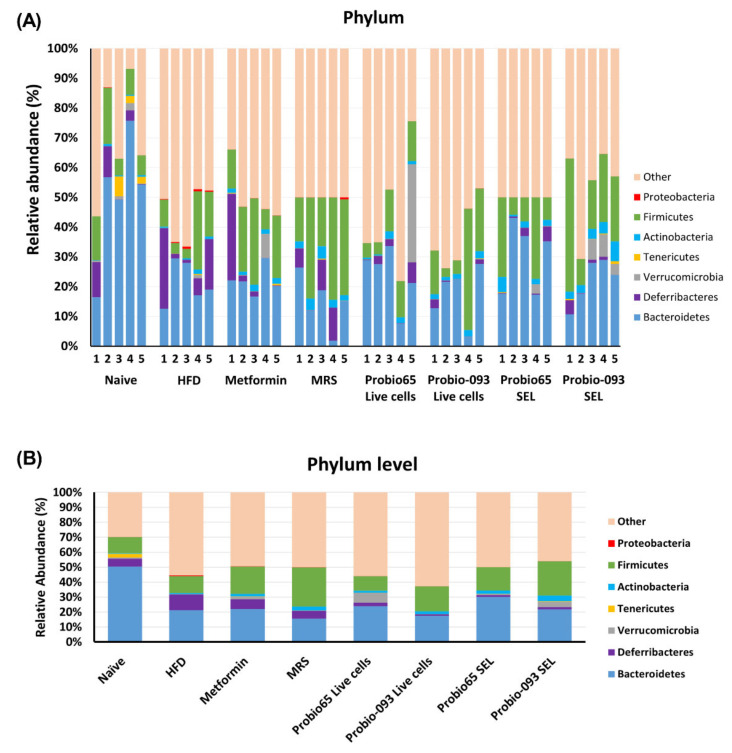
(**A**) The distribution profile of bacterial phylum in each individual mouse and (**B**) Average relative abundance of major phylum in each group. The microbiota profile was determined in mice fecal pellets upon 8 weeks of high-fat diet treatment. Bacterial phylum targeting 16S rRNA was checked with qPCR method. The sequences which could not be clustered in any phylum group are labelled as “others”. Naïve: untreated mice fed with normal diet (*n* = 5); HFD: untreated mice fed with high-fat diet containing 45 kcal% fat (*n* = 5); Metformin: mice on high-fat diet (45 kcal% fat) and gavaged with metformin (0.25 mg/g/day); MRS: mice on high-fat diet (45 kcal% fat) and gavaged with De Man, Rogosa and Sharpe; Probio65 Live cells: mice on high-fat diet (45 kcal% fat) and gavaged with live *Lactobacillus sakei* Probio65 (10⁸ CFU/day); Probio-093 Live cells: mice on high-fat diet (45 kcal% fat) and gavaged with live *Lactobacillus plantarum* Probio-093 (10⁸ CFU/day); Probio65 SEL: mice on high-fat diet (45 kcal% fat) and gavaged with ethanolic extract of *Lactobacillus sakei* Probio65 (5 µL/g/day); Probio-093 SEL: mice on high-fat diet (45 kcal% fat) and gavaged with ethanolic extract of *Lactobacillus plantarum* Probio-093 (5 µL/g/day). Data are represented as ± S.E.M. Statistical analysis was performed with an independent *t*-test.

A total of 6 phyla, namely *Bacteroidetes*, *Firmicutes*, *Actinobacteria*, *Verrucomicrobia*, *Tenericutes*, *Deferribacteres*, and *Proteobacteria*, which represent the dominant gut microbial phyla in mouse, were determined in the fecal samples (Figure 5A). The unknown, which were not categorized in any group, are referred to as “other”.

As exhibited in Figure 5B, at the phylum level, a significant difference was observed in *Deferribacteres*, *Actinobacteria*, and *Proteobacteria*, when treatment groups were related to the HFD group. Mice including in naïve group (*p* = 0.017), *L. sakei* Probio65 Live cells (*p* = 0.007), *L. plantarum* Probio-093 Live cells (*p* = 0.005), *L. sakei* Probio65 SEL (*p* = 0.005), and *L. plantarum* Probio-093 SEL (*p* = 0.008) had lower prevalence of *Deferribacteres* as compared to HFD group; metformin and MRS group had no significant results. The *Actinobacteria* phyla were markedly higher in groups getting *L. plantarum* Probio-093 Live cells (*p* = 0.0087) and *L. plantarum* Probio-093 SEL (*p* = 0.0048). In the case of *Proteobacteria,* a significant difference was not observed in the mice of group naïve, metformin, MRS, and *L. plantarum* Probio-093 Live-cell in comparison with the HFD group. In contrast, mice administered with *L. sakei* Probio65 Live cells (*p* = 0.05), *L. sakei* Probio65 SEL (*p* = 0.05), and *L. plantarum* Probio-093 SEL (*p* = 0.05) had a lesser richness of *Proteobacteria* as correlated to HFD.

### 3.4. Microbiota Composition at the Genus Level

Some genus-level bacteria, including Lactobacillus, Bacteroides, Prevotella, Bifidobacterium, Desulfovibrio, and Clostridium, were also checked.

**Figure 6:**
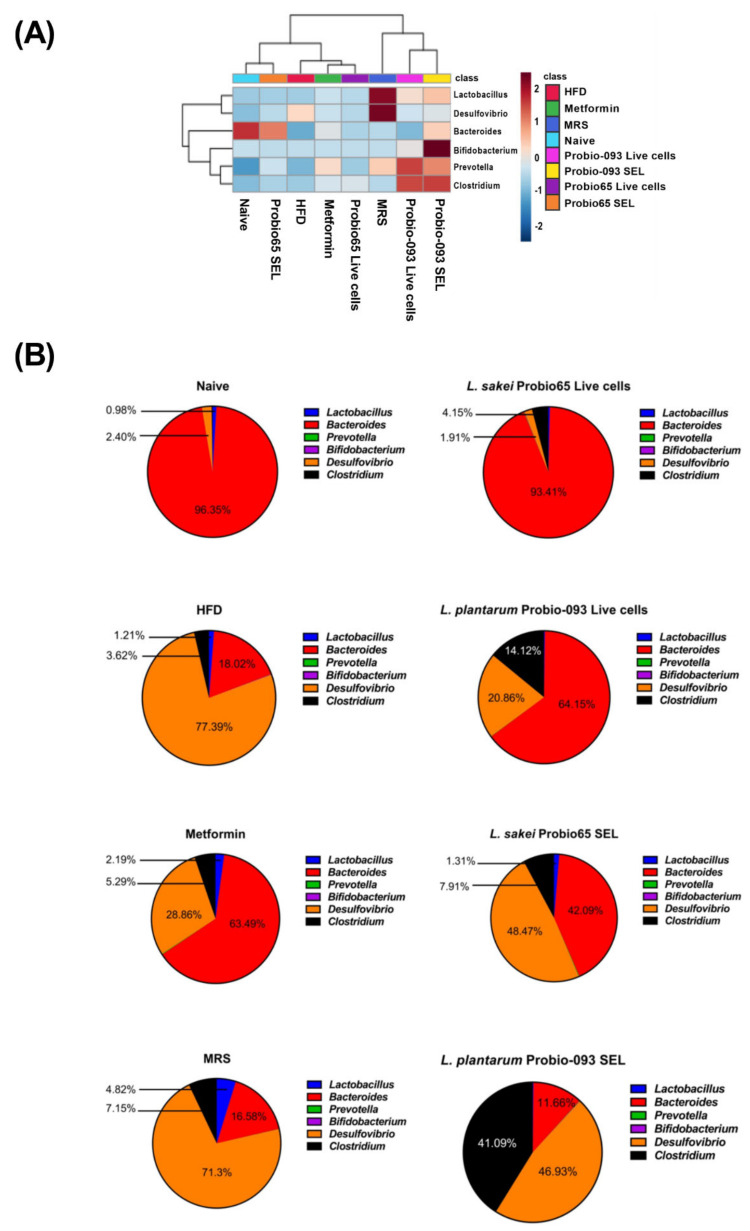
(**A**) Heat map analysis of six dominant genus and (**B**) Genus distribution profile determined in the fecal samples of mice after 8 weeks of high-fat diet treatment. Heat map distinguished abundance of each genus through different color codes with values normalized from 2 to −2; red indicating high abundance and blue showing low abundance. The proportion of predominant bacteria is also presented in the form of pie chart. Naïve: untreated mice fed with normal diet (*n* = 5); HFD: untreated mice fed with high-fat diet containing 45 kcal% fat (*n* = 5); Metformin: mice on high-fat diet (45 kcal% fat) and gavaged with metformin (0.25 mg/g/day); MRS: mice on high-fat diet (45 kcal% fat) and gavaged with De Man, Rogosa and Sharpe; Probio65 Live cells: mice on high-fat diet (45 kcal% fat) and gavaged with live *Lactobacillus sakei* Probio65 (10⁸ CFU/day); Probio-093 Live cells: mice on high-fat diet (45 kcal% fat) and gavaged with live *Lactobacillus plantarum* Probio-093 (10⁸ CFU/day); Probio65 SEL: mice on high-fat diet (45 kcal% fat) and gavaged with ethanolic extract of *Lactobacillus sakei* Probio65 (5 µL/g/day); Probio-093 SEL: mice on high-fat diet (45 kcal% fat) and gavaged with ethanolic extract of *Lactobacillus plantarum* Probio-093 (5 µL/g/day). Mice fecal samples were collected after 8 weeks of treatment. Data are represented as ± S.E.M.

Heat map analysis of bacteria at the genus level showed a distinct microbiota profile having two main clusters, further divided into sub-clusters (Figure 6A). Probio-093 SEL and Probio-093 Live cells were classified into one sub-cluster along with MRS. While naïve and Probio65 SEL were classified into one sub-cluster, the Probio65 live cells (metformin and HFD) shared a more similar profile of microbiota. Based on these observations, the changes in the proportion of bacteria at genus level was determined (Figure 6B). Upon high-fat diet induction for eight weeks, the mice in the HFD group showed a proportionately higher population of *Desulfovibrio* while *Bacteroides* were reduced compared to naïve control. Interestingly, treatments of HFD mice with *L. sakei* Probio65 and *L. plantarum* Probio-093 resulted in a reduced proportion of *Desulfovibrio*, with live cells exerting more notable changes than the SEL extract. Furthermore, the live cells of both strains also favored the growth of *Bacteroides*, especially *L. sakei* Probio65, to the extent where it was similar to naïve control. The strain *L. plantarum* Probio-093 (both live cells and SEL) had a better influence on the growth of *Clostridium* compared to the HFD control.

### 3.5. Microbiota Composition at the Genus Level into Graph Arrangements

**Figure 7:**
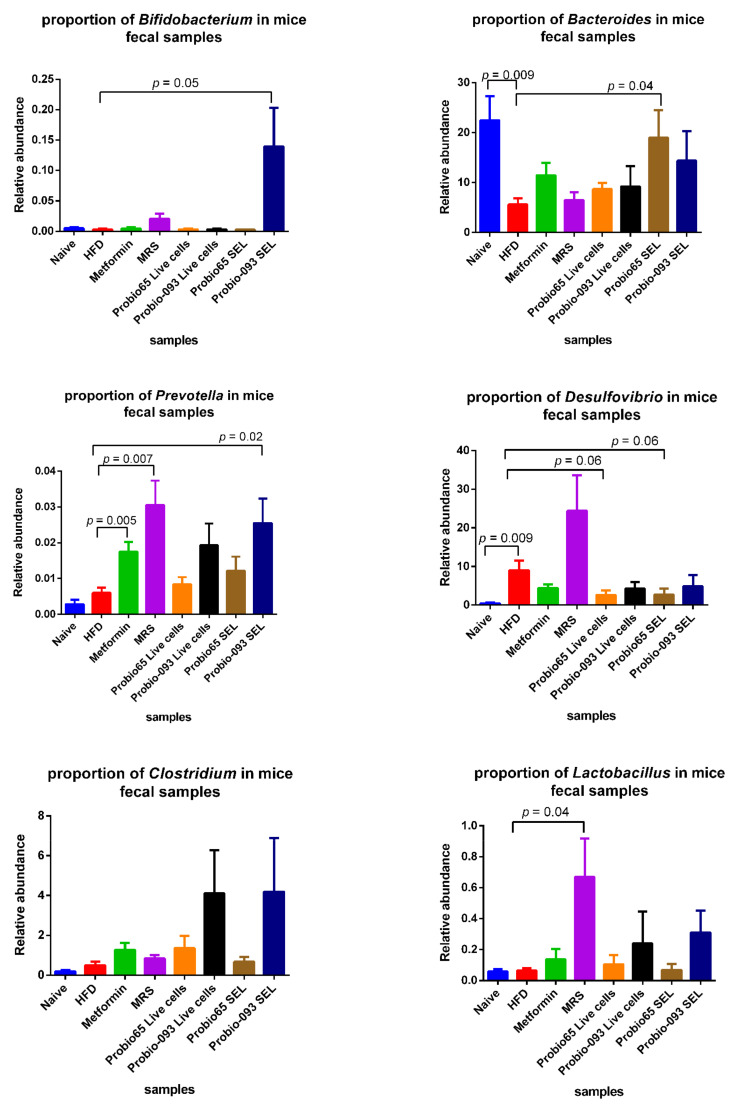
The relative abundance of each genus determined in the fecal samples of mice after 8 weeks of high-fat diet treatment. Naïve: untreated mice fed with normal diet (*n* = 5); HFD: untreated mice fed with high-fat diet containing 45 kcal% fat (*n* = 5); Metformin: mice on high-fat diet (45 kcal% fat) and gavaged with metformin (0.25 mg/g/day); MRS: mice on high-fat diet (45 kcal% fat) and gavaged with De Man, Rogosa and Sharpe; Probio65 Live cells: mice on high-fat diet (45 kcal% fat) and gavaged with live *Lactobacillus sakei* Probio65 (10⁸ CFU/day); Probio-093 Live cells: mice on high-fat diet (45 kcal% fat) and gavaged with live *Lactobacillus plantarum* Probio-093 (10⁸ CFU/day); Probio65 SEL: mice on high-fat diet (45 kcal% fat) and gavaged with ethanolic extract of *Lactobacillus sakei* Probio65 (5 µL/g/day); Probio-093 SEL: mice on high-fat diet (45 kcal% fat) and gavaged with ethanolic extract of *Lactobacillus plantarum* Probio-093 (5 µL/g/day). Mice fecal samples were collected after 8 weeks of treatment. Data are represented as ± S.E.M. Statistical analysis was performed with an independent t-test.

The genera, which showed significant changes upon treatment, are depicted in Figure 7. Among all, the mice group treated with *L. plantarum* Probio-093 SEL had significantly more affluence of *Bifidobacterium* as a concern with the HFD mice group (*p* = 0.05). The *Bacteroides* proportion was higher in the naive group of mice (*p* = 0.009) and those treated with *L. plantarum* Probio-093 (*p* = 0.04). The *Prevotella* abundance was significantly high in mice administered with metformin (*p* = 0.005), MRS (*p* = 0.007), and *L. plantarum* Probio-093 SEL (*p* = 0.02) when compared with the HFD group. In the naive group, in comparison with the HFD group, the *Desulfovibrio* was noticeably lower (*p* = 0.009). In contrast, *Desulfovibrio* was marginally decreased in those mice treated with *L. sakei* Probio65 Live cells (*p* = 0.06) and *L. sakei* Probio65 SEL (*p* = 0.06). Compared to the treatment group with the high-fat diet group, the relative abundance of *Lactobacillus* was enhanced in the mice receiving MRS (*p* = 0.04). In the case of the *Clostridium* genus, no difference was observed in any of the groups.

## 4. Discussion

Live cells and SEL of both *L. sakei* Probio65 and *L. plantarum* Probio-093 exhibited inhibition of α-GLU and α-amylase with different concentrations, being more effective than acarbose, a reference inhibitor of these enzymes. α-amylase and α-GLU enzymes played significant role in the management of blood glucose, and thus are closely associated with the development of diabetes. Currently, the inhibitors of these enzymes such as acarbose are accepted as anti-diabetic and anti-obesity drugs [35]. α-GLU and α-amylase carry out the hydrolysis of complex carbohydrates and starch, producing monosacharrides in the small intestine [50].

**Figure 8:**
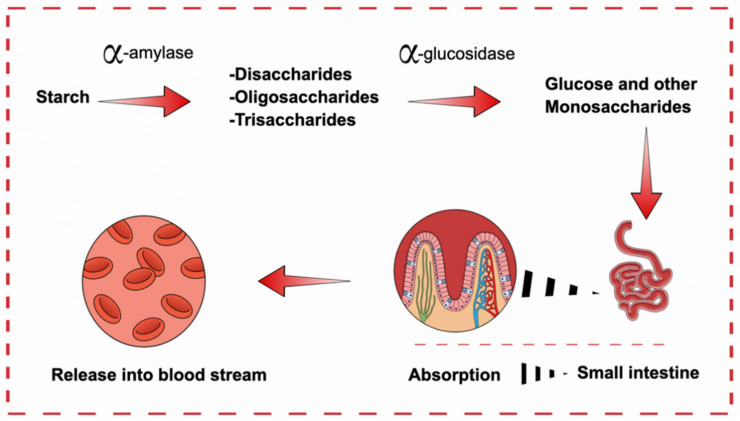
Diagram illustrating the release of glucose into blood stream from small intestine. First α-amylase converts starch into disaccharides. These oligosaccharides are then converted to monosaccharides by α-GLU.

During the digestive process, starches are first hydrolyzed by α-amylase to disaacharides, oligosaccharides, and trisaccharides. Subsequently, α-GLU converted these oligosaccharides into monosaccharides (mainly glucose) which are eventually absorbed into the intestine and assimilated into the blood from the intestine (Figure 8). Blood carries these monosaccharides to individual cells where the cell machinery converts them to energy with the aid of insulin. Without proper recognition of insulin by the cells or controlled secretion of insulin by the pancreas, the monosaccharides will not be utilized as energy and remains in the blood. This in turn raises blood glucose levels, leading to hyperglycemia. Therefore, the hydrolysis of complex carbohydrates by the enzymes α-amylase and α-GLU situated in the brush border of jejunum will be the main source of increased blood sugar level. As such, the enzymes are closely associated with the risk of T2D. In current medical practice, α-amylase and α-GLU are essential targets for T2D and their inhibitors are used as drugs to alleviate the disease. By delaying the absorption of carbohydrates from the small intestine, blood glucose level is controlled, so is the progression of T2D [51].

Two strains, namely *L. sakei* Probio65 and *L. plantarum* Probio-093, were first screened for enzyme inhibition to determine the anti-diabetic potential of the strains. As these two strains successfully showed potent inhibition towards the enzyme (α-amylase and α-GLU), on par with standard acarbose, the authors are interested in validating further the effect of these strains in an animal study. In subsequent analysis (in-vivo), we used 4 week old C57BL/6J male mice fed with 45% kcal fat which turned the mice obese and later developed a risk of T2D. Consistently, HFD-mice treated with the strain *L. plantarum* Probio-093 showed a reduction in blood glucose level, justifying its biologically functional activity in-vivo. The reduction of blood glucose level correlated directly with the inhibition of these enzymes.

Mice were treated with ethanol extract and live cells of *L. sakei* Probio65 and *L. plantarum* Probio-093 for 8 weeks. Gut microbiota was analyzed upon 8 weeks of treatment using the qPCR method. Gut microbiota contributes to human health and is considered a basic part of the human body. Changes in the human body are linked to shifts in gut bacteria. In this study, we utilized various phylum and genus-specific primers to detect the shifts in the gut microbiota of mice treated with probiotics *L. sakei* Probio65 and *L. plantarum* Probio-093. Probiotics restored a prominent amount of gut microbiome in a commendable way. They may introduce beneficial functions to gut microbial communities, resulting in amelioration or prevention of gut inflammation and other intestinal or systemic disease phenotypes.

In our findings, phylum *Deferribacteres* and *Proteobacteria* were high in abundance in the HFD group. In previous studies, *Deferribacteres* were also reported to elevate in the gut microbiome of HFD-induced obesity [52]. Expanding evidence suggests that *Deferribacteres* positively correlate with pro-inflammatory cytokines, caused due to aggravation of inflammation [53]. HFD increases adiposity of visceral adipose tissues [54]. At the time of increased nutrients in the body, if fat cells cannot extend quickly enough to accommodate the increased fatty acid flow, the excess released fatty acids will accumulate in other tissues, such as the liver and skeletal muscles, triggering lipotoxicity and increasing systemic insulin resistance [55,56]. In the result of aberrant metabolic functions, hyperglycemia increase, and insulin sensitivity decrease, consequently, stimulate additional inflammatory responses in obesity and promote obesity-induced metabolic disease T2D [57]. All our treatment groups reduce the *Deferribacteres*, which also means the treatment helps to eliminate pathogenic microbial community, which can aggravate inflammation that may lead to diabetes.

The lower proportion of *Proteobacteria* in probiotics treated groups *L. sakei* Probio65 Live cells and *L. plantarum* Probio-093 SEL and Live cells are beneficial because these bacteria (*Proteobacteria*) are known for their lipopolysaccharide (LPS) production [58]. LPS production is a risk for the development of T2D. LPS activates the toll-like receptor 4 (TLR4) signal path to trigger the section of pro-inflammation cytokines, including interleukin (IL)-6, IL-17, and tumor necrosis factor (TNF)-α [59,60]. In accordance, LPS is perused by LPS binding-protein (LBP) in the blood and shifted to the TLR4 receptor complex. LPS joining to TLR4 retain intracellular adaptor proteins, which directs to the activation of inflammatory kinases integrated with insulin impedance, inclusive of the mitogen-activated protein kinases (MAPK) [c-Jun N-terminal kinase (JNK), p38, and extracellular-signal related kinase (ERK)], and the I kappa B kinase (IKK) complex. Downstream transcription factors such as nuclear factor κB (NFκB) and activator protein-1 (AP-1) upraise the expression of inflammatory proteins [61]. Moreover, inflammation and insulin resistance can prevent by distorted expression of TLR4, in response to severe variations in dietary fat [62].

The *Actinobacteria* phyla were significantly higher in mice administered with probiotic SEL and Live cells of *L. plantarum* Probio-093. *Actinobacteria* have potential as important new sources of enzyme inhibitors. Imada (2005) reported different types of enzyme which were inhibited, like α-glucosidase, N-acetyl-b-Dglucosaminidase, pyroglutamyl peptidase, and α-amylase [63]. Enzyme retarders are valuable in controlling carbohydrate-dependent diseases such as diabetes, obesity, hyperlipidemia, and melanogenesis in the skin [64]. Moreover, abdominal obesity is commonly observed in aging and the *Actinobacteria* is diminished as an individual grows older, signifying the metabolic health-promoting effect of *Actinobacteria* [65]. Besides, to quantify bacteria at the phylum, some genus level bacteria were also observed.

In our results, treatment group *L. plantarum* Probio-093 SEL has an elevated number of *Bifidobacterium*. *Bifidobacterium* is advantageous in lactose intolerance, bile resistance, eczema, antibiotic-associated diarrhea, cholesterol-lowering abilities, and immune system improvement [66,67,68,69]. Two studies ensured that changes in gut microbiota are possible to recognize after weight gain even in early stages, as they conducted results on overweight children. They signified a decrease of good bacteria with obesity, such as *Bifidobacteria* and *Akkermansia muciniphila*-like bacteria, allied with an excess of pathogens or gram-negative bacteria, such as *Enterobacteriaceae* and *Staphylococcus aureus* [70,71]. Many functional ingredients make up by bacterial genus Lactobacillus and Bifidobacterium are present in the vast majority of commercialized probiotics food.

We have presented that feeding mice with a HF diet decreased the dominant members of the mouse intestinal microbiota—*Bacteroides* and *Prevotella.* In contrast, metformin and *L. plantarum* Probio-093 SEL administered mice increased *Bacteroides* and *Prevotella*. *Prevotella* species were demonstrated to be positively associated with propionate production, that has significant roles in forestalling weight gain, by lessening serum cholesterol and diminishing hepatic lipogenesis [72].

Our data showed that *Desulfovibrio* abundance was marginally lower in probiotic *L. sakei* Probio65 SEL and live cells treated mice while *Bacteroides* were more consequential in those mice treated with *L. sakei* Probio65 SEL than in HFD induced diabetes mice. *Bacteroides* are an important group of bacteria that produce SCFA, and SCFA helps in maintaining homeostasis of the gut and are important contents of the metabolism mechanism [73]. Besides, insulin, one of the potent anti-diabetes dietary supplements, was found to promote *Bacteroides*, *Lachnospiraceae*, and *Phascolarctobacterium*, in the diabetic mouse. Knockdown of mediators of biological effects of SCFA and SCFA receptors (which are expressed on the cell surface) leads to inflammation, glucose intolerance, and diet-induced obesity in mice [74]. On the other hand, the expansion of *Desulfovibrio* is highly associated with metabolic syndrome and obesity [75]. *Desulfovibrio* is a member of gram-negative bacteria, which are promoters of LPS and hence deface the gut barrier [76,77,78]. HFD can generate a leaky gut and cause bacterial lysis, permitting the LPS of Gram-negative bacteria to enter the enterohepatic circulation [79]. LPS can initiate pro-inflammatory cytokine formation, directing to impaired insulin reactivity and inauguration of insulin resistance-related metabolic disorders [80].

The mucosal surface is one of the first-line defense components of the innate immune system, where most microbe-host interactions occur [81]. Disruption of this barrier results in dysregulated gut permeability. A gut barrier might be damaged by HFD, in the obesity-induced diabetic model, and lead to translocation of bacteria and leak of harmful substances like LPS produce by pathogenic bacteria. Based on our data, we postulate that supplementation of *L. sakei* Probio65 and *L. plantarum* Probio-093 to HFD-induced diabetic mice might adjust the gut barrier by decreasing LPS, develop the immune response, and defend in opposition of threatening bacteria, ensuing the mitigation of intestinal inflammation and release essential along with helpful substrates in metabolism pathway through modulating gut microbiota. Results of our study approved *L. sakei* Probio65 and *L. plantarum* Probio-093 are worthy to reduce obesity-linked T2D by progressing the gut microbiota.

## 5. Conclusions

These data support a pivotal role of probiotics *L. sakei* Probio65 and *L. plantarum* Probio-093 in the pathogenesis of obesity-related T2D disorder. In summary, our study reinforces the hypothesis that *L. sakei* Probio65 and *L. plantarum* Probio-093 have the potential to inhibit target enzymes that are associated with diabetes. *L. sakei* Probio65 and *L. plantarum* Probio-093 also significantly alleviated HFD-related diabetes in mice by modulating gut microbiota profile intensely related to metabolic disease. Among all four treatment groups (*L. sakei* Probio65 Live cells, SEL, and *L. plantarum* Probio-093 Live cells and SEL), the SEL of *L. plantarum* Probio-093 is showing the most potent effect. The beneficial effects are contributed by the enhancement of gut microbiota profile strongly linked to metabolic disease.

## Figures and Tables

**Table 1:** Formulation of experimental diets Rodent Diet with 45 kcal% Fat (D12451).

Product Ingredients	g%	kcal%
Protein	24	20
Carbohydrate	41	35
Fat	24	45
Total	89	100
kcal/g	4.73	
Casein, 30 Mesh	200	800
L-Cystine	3	12
Corn Starch	72.82	91
Maltodextrin 10	100	400
Sucrose	172.8	691
Cellulose, BW 200	50	0
Soybean Oil	25	225
Lard	177.5	1598
Mineral Mix S10026	10	0
DiCalcium Phosphate	13	0
Calcium Carbonate	5.5	0
Potassium Citrate, 1 H_2_O	16.5	0
Vitamin Mix V10001	10	40
Choline Bitartrate	2	0
FD&C Red Dye #40	0.05	0
FD&C Blue Dye #1	0	0
FD&C Yellow Dye #5	0	0
Total	858.15	4057

**Table 2:** Oligonucleotides used in this study for qPCR analyses.

Target Primers	Sequence (5-3)	Tm (°C)	References
*Bacteroidetes*	(F) GGARCATGTGGTTTAATTCGATGAT		
	(R) AGCTGACGACAACCATGCAG	58	[40]
*Firmicutes*	(F) GGAGYATGTGGTTTAATTCGAAGCA5		
	(R) AGCTGACGACAACCATGCAC	59.7	[40]
*Actinobacteria*	(F) TGTAGCGGTGGAATGCGC		
	(R) AATTAAGCCACATGCTCCGCT	58.2	[41]
*Verrucomicrobia*	(F) TCAKGTCAGTATGGCCCTTAT		
	(R) CAGTTTTYAGGATTTCCTCCGCC	55.9	[41]
*Tenericutes*	(F) ATGTGTAGCGGTAAAATGCGTAA		
	(R) CMTACTTGCGTACGTACTACT	57	[41]
*Deferribacteres*	(F) CTATTTCCAGTTGCTAACGG		
	(R) GAGHTGCTTCCCTCTGATTATG	55.2	[41]
*Proteobacteria*	(F) CATTGACGTTACCCGCAGAAGAAGC		
	(R) CTCTACGAGACTCAAGCTTGC	59.8	[42]
*Lactobacillus*	(F) AGCAGTAGGGAATCTTCCA		
	(R) CACCGCTACACATGGAG	54.5	[43]
*Bacteroides*	(F) CGATGGATAGGGGTTCTGAGAGGA		
	(R) GCTGGCACGGAGTTAGCCGA	54.5	[44]
*Prevotella*	(F) CACCAAGGCGACGATCA		
	(R) GGATAACGCCYGGACCT	55.1	[45]
*Bifidobacteria*	(F) GCGTGCTTAACACATGCAAGTC		
	(R) CACCCGTTTCCAGGAGCTATT	60.2	[46]
*Desulfovibrio*	(F) CCGTAGATATCTGGAGGAACATCAG		
	(R) ACATCTAGCATCCATCGTTTACAGC	62.9	[47]
*Clostridium*	(F) AAATGACGGTACCTGACTAA		
	(R) CTTTGAGTTTCATTCTTGCGAA	53.2	[48]
Universal	(F) AAACTCAAAKGAATTGACGG		
	(R) CTCACRRCACGAGCTGAC	51.1	[49]

**Table 3:** Amplification efficiency of phylum and genus specific primers by qPCR.

Target Primers	Efficiency (%)
*Bacteroidetes*	101.7
*Firmicutes*	100.5
*Actinobacteria*	101.3
*Verrucomicrobia*	95.2
*Tenericute*	105.3
*Deferribacteres*	97
*Proteobacteria*	101.7
*Lactobacillus*	93.4
*Bacteroides*	104.4
*Prevotella*	101.7
*Bifidobacteria*	101.7
*Desulfovibrio*	93.4
*Clostridium*	94.9
Universal	91.6

## Data Availability

The data presented in this study are available on request from the corresponding author. The data are not publicly available due to patent rights.
